# Genome-Wide Analysis of Salicylate and Dibenzofuran Metabolism in *Sphingomonas Wittichii* RW1

**DOI:** 10.3389/fmicb.2012.00300

**Published:** 2012-08-23

**Authors:** Edith Coronado, Clémence Roggo, David R. Johnson, Jan Roelof van der Meer

**Affiliations:** ^1^Department of Fundamental Microbiology, University of LausanneLausanne, Switzerland; ^2^Department of Environmental Systems Science, Swiss Federal Institute of Technology ZürichZurich, Switzerland; ^3^Department of Environmental Microbiology, Swiss Federal Institute of Aquatic Science and TechnologyDübendorf, Switzerland

**Keywords:** polycyclic aromatic hydrocarbons, bioremediation, transposon screening, microarray analysis

## Abstract

*Sphingomonas wittichii* RW1 is a bacterium isolated for its ability to degrade the xenobiotic compounds dibenzodioxin and dibenzofuran (DBF). A number of genes involved in DBF degradation have been previously characterized, such as the *dxn* cluster, *dbfB*, and the electron transfer components *fdx1*, *fdx3*, and *redA2*. Here we use a combination of whole genome transcriptome analysis and transposon library screening to characterize RW1 catabolic and other genes implicated in the reaction to or degradation of DBF. To detect differentially expressed genes upon exposure to DBF, we applied three different growth exposure experiments, using either short DBF exposures to actively growing cells or growing them with DBF as sole carbon and energy source. Genome-wide gene expression was examined using a custom-made microarray. In addition, proportional abundance determination of transposon insertions in RW1 libraries grown on salicylate or DBF by ultra-high throughput sequencing was used to infer genes whose interruption caused a fitness loss for growth on DBF. Expression patterns showed that batch and chemostat growth conditions, and short or long exposure of cells to DBF produced very different responses. Numerous other uncharacterized catabolic gene clusters putatively involved in aromatic compound metabolism increased expression in response to DBF. In addition, only very few transposon insertions completely abolished growth on DBF. Some of those (e.g., in *dxnA1*) were expected, whereas others (in a gene cluster for phenylacetate degradation) were not. Both transcriptomic data and transposon screening suggest operation of multiple redundant and parallel aromatic pathways, depending on DBF exposure. In addition, increased expression of other non-catabolic genes suggests that during initial exposure, *S. wittichii* RW1 perceives DBF as a stressor, whereas after longer exposure, the compound is recognized as a carbon source and metabolized using several pathways in parallel.

## Introduction

Dibenzofuran (DBF) and dibenzo-*p*-dioxin (DBD) are poorly water soluble polycyclic aromatic hydrocarbons (PAH) that are formed as byproducts of coal tar industrial processes, during incineration, and in paper pulp bleaching. DBF, DBD, and related compounds with chlorine substitutions are widely present at low concentrations in the environment and the food chain (Bowes et al., 1973; Buser et al., 1985; Beck et al., 1994; Johansen et al., 1996), and detrimental effects of exposure have been reported (Zitko et al., 1973; Yoshihara et al., 1981; McNulty, 1985; Pluim et al., 1993; Soong and Ling, 1997). A number of bacteria can use DBF and DBD as sole carbon and energy sources, including *Staphylococcus auriculans* (Monna et al., 1993), *Nocardioides aromaticivorans* (Kubota et al., 2005), *Rhodococcus* sp. HA01 (Aly et al., 2008), *Pseudomonas putida* B6-2 (Li et al., 2009), *Sphingomonas yanoikuyae* B1 (Cerniglia et al., 1979), *Sphingomonas* sp. HH69 (Fortnagel et al., 1990), *Sphingomonas* sp. XLDN2-5 (Gai et al., 2007), *Sphingomonas* sp. RW16 (Wittich et al., 1999), or *S. wittichii* RW1 (Wittich et al., 1992; Wilkes et al., 1996). Consequently, there has been an interest to apply such isolates for bioaugmentation of DBF/DBD-contaminated sites. Megharaj et al. (1997) and Halden et al. (1999) observed DBF and DBD degradation by *S. wittichii* RW1 applied to inoculated soil microcosms, while Aso et al. (2006) reported increased DBF degradation rates by inoculating a modified strain of *S. wittichii* RW1 in contaminated soil.

Despite such anecdoctal reports, bioaugmentation with strains such as RW1 have not consistently resulted in accelerated pollutant degradation rates. Possible reasons that have been put forward to explain the limited success of bioaugmentation include the lack of essential nutrients in the soil, the creation of toxic dead-end products (Halden et al., 1999), the low availability of the hydrocarbons (Aso et al., 2006), and the poor ability of the bacteria to adapt to the soil environment (Megharaj et al., 1997). However, the cellular responses of bacteria such as RW1 to different conditions have not been extensively studied, and perhaps DBF/DBD metabolism is regulated in a complex manner depending on the types of exposure.

To address this knowledge gap, we systematically explored gene transcription of *S. wittichii* RW1 at a genome-wide level in batch and chemostat cultures, with and without exposure to DBF or salicylate (SAL) under different cellular growth and environmental conditions. *S. wittichii* RW1 (Wittich et al., 1992) is a gram-negative α-*Proteobacterium*, with a genome consisting of one chromosome and two mega plasmids, named pSWIT01 and pSWIT02 (Miller et al., 2010). The larger mega plasmid pSWIT01 has been reported to be similar to pNL1 from *Novosphingobium aromaticivorans*, whereas the smaller pSWIT02 contains a number of genes previously implicated in DBF/DBD degradation (Bunz and Cook, 1993; Bünz et al., 1993; Armengaud and Timmis, 1998; Armengaud et al., 1998, 1999, 2000; Basta et al., 2004; Miller et al., 2010). Several enzymes participating in DBF degradation have been purified and characterized, notably the initial multicomponent DBF dioxygenase, which is composed of the terminal oxidase DxnA1, a reductase (RedA2), and a ferredoxin (Fdx1; Bunz and Cook, 1993). In addition, a 2,2′,3-trihydroxybiphenyl dioxygenase activity (DbfB) and its corresponding gene (Swit_4902) were characterized (Happe et al., 1993), and two hydrolases, DxnB and DxnB2 (Swit_4895 and Swit_3055), were described (Bünz et al., 1993; Seah et al., 2007). Several of the genes for the above-mentioned enzymes were cloned and characterized, such as *fdx1* (Swit_5088, Armengaud and Timmis, 1997), *redA2* (Swit_4920, Armengaud and Timmis, 1998), and *dxnA1A2B* (Swit_4897 and Swit_4896, Armengaud et al., 1998). The expression of *dxnA1* in RW1 was reported to be higher in DBF-grown than in LB-grown cells (Armengaud et al., 1998), while Bunz and Cook (1993) observed that DxnA1 was constitutively synthesized in RW1 cells growing on acetate, benzoate, or SAL as sole carbon and energy source. Finally, a number of other genes, notably the gene *fdx3* (Swit_4893) and a cluster of genes named *dxnCDEFGHI* (Swit_4887–4894) were cloned and overexpressed in *Escherichia coli*. Their enzyme activities were determined, suggesting that they are involved in DBF degradation by RW1 (Armengaud et al., 1999).

The main objective of the study presented here was to study RW1 gene transcription at the genome-wide level during exposure to DBF. Our hypothesis was that we would clearly see the expression of the above-mentioned genes in DBF degradation, but perhaps would also detect other genes for catabolic functions specifically induced or repressed during exposure to DBF. Secondly, we were interested to study how the expression of RW1 genes would change under different growth conditions and exposures to DBF, which might give us clues about how expression in the DBF degradation pathway changes under environmental conditions. Because DBF is poorly water soluble (∼5 mg/l) we followed pathway induction by exposing RW1 cells in batch culture to carbon substrate exchange or by transiting stably chemostat-growing cultures from medium without to medium with saturating DBF levels. Finally, we cultured cells in batch culture with DBF, SAL, or phenylalanine (PHE) as sole carbon and energy source. A custom-made RW1 microarray (Johnson et al., 2011) was used to analyze differences in genome-wide gene expression under different conditions. In addition, we used genome-wide transposon screening (Langridge et al., 2009) to further identify genes necessary for growth on DBF as sole carbon and energy source.

## Materials and Methods

### Bacterial cultivation

A stock of *S. wittichii* RW1 was kept at −80°C and a small aliquot was plated on minimal medium agar (1.5% w/v) with 5 mM SAL, or by placing DBF crystals in the lid of a Petri dish. Liquid cultures were always prepared from an isolated colony of RW1 from a plate. Minimal media (MM) were based on DSM457 media (Johnson et al., 2011) amended with 5 mM SAL, 5 mM PHE, or with DBF crystals in a dosage of 5 μmol/ml as carbon source. All cultures were incubated at 30°C with rotary shaking at 180 rpm. Batch cultures of strain RW1 were prepared in 50 ml Erlenmeyer flasks containing 15 ml of MM. The cultures were started at an initial culture turbidity of 0.005 (at 600 nm, OD_600_) for all carbon sources evaluated. Carbon-limited continuous culturing of *S. wittichii* RW1 was carried out in triplicate 500-ml working volume reactors (Infors-HT), containing 400 ml MM and 5 mM PHE as carbon and energy source. Reactors were inoculated with 100 ml of a preculture of *S. wittichii* RW1, which was prepared by inoculating a single colony into a 1 l Erlenmeyer flask with 300 ml of MM plus 5 mM PHE and streptomycin (Sm), and grown until stationary phase (OD_600_ ∼ 1). Streptomycin was added (at 50 μg/ml) to the growth media to avoid culture contamination, since strain RW1 is naturally resistant to Sm. The triplicate fermentors were then first operated in batch mode at 30°C and with a stirring speed of 200 rpm. The pH and partial oxygen pressure (pO_2_) were monitored online and were maintained at 7 and ∼90%, respectively. When the culture turbidity reached a steady value of OD_600_ = 1 (the maximum OD obtained with 5 mM PHE), the inflow of fresh MM with 5 mM PHE was started. The flow rate was set to 23.8 ml/h, giving a dilution rate of 0.05 h^−1^, which corresponds to a doubling time of 14.6 h. The culture turbidity was measured at regular time intervals and culture samples were plated on LB agar without Sm to verify culture purity. Silicon based antifoam suspension (Antifoam-B emulsion, Sigma–Aldrich) was added at a rate of 0.025 ml/h to avoid excessive culture foaming. The continuous culture was considered in equilibrium after five reactor volume changes and when the measured parameters (culture turbidity and pO_2_) were stable.

### Genome-wide transposon screening

A transposon insertion library was constructed by conjugating plasmid pRL27 from *E. coli* BW20767 to *S. wittichii* RW1 as described by Larsen et al. (2002). RW1 transconjugants were selected on MM + SAL plates in the presence of 50 μg/ml kanamycin. RW1 kanamycin-resistant colonies were pooled and stored in aliquots at −80°C in the presence of 15% (v/v) glycerol. Individual aliquots of the library were then grown for five passages in batch culture on MM with 5 mM SAL or with DBF crystals (∼50 generations). After five subsequent transfers the total genomic DNA was extracted from the cultures and from the initial library. Aliquots of 5 μg DNA were fragmented and used for Illumina library preparation, during which specific DNA fragments containing the transposon DNA were amplified, as described in Langridge et al. (2009). Samples were then sequenced on an Illumina HiSeq2000 machine at the Lausanne Genomic Technologies Facility (University of Lausanne). Transposon containing sequences were filtered, trimmed, and mapped on the *S. wittichii* genome using the *Xpression* pipeline, which was developed by the Harwood lab at the University of Washington^1^. Genes without any transposon insertions were scored and compared between the two conditions and the starting library.

### Short exposure of *S. wittichii* RW1 to DBF in batch cultures

*Sphingomonas wittichii* RW1 was grown in MM + PHE to stationary phase and this culture was used to inoculate eight identical replicate 100 ml Erlenmeyer flasks with 20 ml of MM + PHE. These cultures were then grown to exponential phase (OD_600_ ∼ 0.2), upon which the cells were transferred to eight replicate 50 ml glass centrifuge tubes and centrifuged at 6000 rpm for 2 min. The supernatant was discarded and 20 ml of preheated (30°C) MM + PHE was added to four replicate tubes, whereas 20 ml of preheated (30°C) MM + DBF (presaturated) was added to the other four replicate tubes. Cells were resuspended immediately and the tubes were incubated in a rotary shaker at 150 rpm for 30 min at 30°C. After this period, the cells were collected by filtering the cultures over a 0.2 μm Millipore filter, which were then transferred into 2 ml tubes and immediately frozen in liquid N_2_. The cells on filters were stored at −80°C until further processing for RNA isolation.

### Long exposure of *S. wittichii* RW1 to DBF in batch cultures

Secondly, we tested continuous exposure of *S. wittichii* RW1 in batch culture to DBF compared to PHE. A single stationary phase preculture grown in MM + PHE was used as inoculum for two sets of four replicate cultures in 100 ml Erlenmeyer flasks with 20 ml of either MM plus 5 mM PHE or MM plus saturating DBF (dosage of 5 μmol/ml). Cultures were grown until an OD_600_ of 0.2, after which the cells were collected on 0.2 μm filters (Millipore), immediately transferred into 2 ml tubes and frozen in liquid N_2_. The filters were kept at −80°C until further processing for RNA isolation.

### Transient exposure of *S. wittichii* RW1 to DBF in continuous culture

In order to achieve an immediate pulse addition of DBF to *S. wittichii* RW1 cells which otherwise experienced carbon-limited conditions, we used cells growing continuously on MM + PHE. Triplicate fermentors with stably growing RW1 cultures on MM + PHE were induced with DBF by injecting a volume of 1 ml DBF dissolved in 2,2′,4,4′,6,8,8′-heptamethylnonane (HMN, at 2.5 mg/ml) into each fermentor (500 ml culture volume) and subsequently changing immediately the inflow medium to MM plus 5 mM PHE plus saturated amounts of DBF (crystals visible in the stirred medium). Addition of HMN to exponentially growing cultures on PHE did not cause any growth rate retardation (not shown). Culture samples (4 × 2 ml) were taken, transferred to 2 ml centrifuge tubes with screw cap and centrifuged at 13,000 rpm for 1 min at room temperature. The supernatant was quickly removed by decanting and the cell pellets were frozen with liquid N_2_. Control samples (*T*_0_) were taken immediately before the transition. Further samples were taken 30 min, 1, 2, and 6 h after the transition to medium with DBF. The cell pellets were stored at −80°C until further processing for RNA isolation.

### RNA processing, microarray hybridization, and analysis

RNA was extracted and purified from the frozen cells on the filters or cell pellets by hot-phenol extraction as described elsewhere (Johnson et al., 2008). cDNA was labeled from the total RNA pool using reverse transcription in the presence of cyanine-3-dCTP. A total of 60 ng of cDNA were used to hybridize to an Agilent 8 × 15 K custom *S. wittichii* RW1 microarray (Johnson et al., 2011). The microarray contains a total of 12,873 50-mer probes that cover >99% of the predicted protein coding genes (5323 out of 5345) within the genome of strain RW1. One 8 × 15K slide contains eight replicate microarrays. The cDNA samples from quadruplate biological replicates and two experimental conditions (e.g., long exposure to DBF versus long exposure to PHE), were positioned randomly on the eight replicate microarrays of a slide to eliminate slide-to-slide variations. Slides were hybridized and scanned following the One-color Microarray-Based Gene Expression Analysis Manual (Agilent Technologies, Santa Clara, CA, USA) protocols. Signals were extracted using Agilent Feature Extraction software 10.5.1.1 (Agilent). Microarray data were quantile normalized and globally scaled using GENE SPRING GX software (version11). *p*-Values were then first calculated on gene set comparisons between treatment and control conditions using the Welch’s *t*-test with unequal variances. *p*-Values were then corrected in the Benjamini and Hochberg procedure for multiple hypothesis testing and converted into false discovery rates (FDRs). For a gene to be classified as differentially expressed between two conditions the FDR had to be less than 0.05 and the fold-difference in average hybridization signal intensities from biological replicates had to be greater than two (Johnson et al., 2008, 2011). All microarray data sets have been deposited as a single file in the NCBI Gene Expression Omnibus under accession number GSE37328 according to MIAME standards^2^. Genome-wide gene expression data of S. *wittichii* RW1 in batch culture growing exponentially on SAL were extracted from accession number GSE26705, and used to compare to those of PHE (SAL/PHE, see Table 1) and DBF batch grown cells (SAL/DBF, Table 1), using the outlined GENESPRING procedure above.

**Table 1:** **RW1 gene cluster representatives for putative aromatic compound metabolism with significantly changed expression levels or proportional abundances in transposon libraries**.

Cluster^1^	Swit Locus	Name or gene name	Strand	Tn library^2^			Expression, fold change^3^							
				TN01	SAL	DBF	SAL/PHE	SAL/DBF	DBF/PHE	DBF shock	Chemostat shift			
											30 m	1 h	2 h	6 h
310–312	311	Carboxymuconolactone decarboxylase	**<**	**−(+)**	**−(±)**	**−**			1.21	1.93	2.81	2.51	2.03	1.25
748-749	749	Enoyl-CoA hydratase *PaaG*	**<**	**−(−)**	**−(−)**	**+**	**0.04**		0.72	**0.12**	0.31	0.35	0.35	0.31
750–758	750	3-Hydroxyacyl-CoA dehydrogenase	**>**	**+(−)**	**−(−)**	**−**	**0.07**		0.53	**0.11**	0.48	0.47	0.38	0.48
	753	*paaA*	**>**	**+(+)**	**−(±)**	**+**	**0.04**		0.50	**0.04**	0.24	**0.15**	**0.14**	**0.16**
	754	*paaB*	**>**	**+(+)**	**−(±)**	**±**	**0.02**		**0.24**	**0.05**	**0.14**	**0.07**	**0.05**	**0.05**
	755	*paaI*	**>**	**−(−)**	**−(−)**	**−**	**0.02**		**0.19**	**0.05**	**0.17**	**0.10**	**0.05**	**0.06**
	756	*paaJ*	**>**	**+(+)**	**−(±)**	**−**			1.06	1.66	1.72	1.51	1.45	1.01
975–978	975	Muconate cycloisomerase	**>**	**±(+)**	**+(+)**	**±**	**8.36**		1.72	1.06	0.74	0.58	0.46	1.95
	978	3-Oxoadipate enol-lactonase	**>**	**+(+)**	**+(+)**	**±**	**2.26**	**2.20**	1.02	1.56	1.33	1.05	0.60	1.04
1639–1644	1639	4-Oxalocrotonate decarboxylase	**>**	**+(−)**	**−(−)**	**−**			1.68	1.17	0.99	1.13	1.47	1.83
	1643	FMN-dependent alpha-hydroxy acid dehydrogenase	**>**	**±(+)**	**±(±)**	**−**			1.27	0.92	1.13	1.08	1.10	1.05
1754–1760	1757	Rieske-type protein; beta subunit	**>**	**−(+)**	**±(±)**	**−**			0.95	0.74	0.96	0.86	0.81	0.80
	1759	Ferredoxin	**>**	**+(±)**	**−(−)**	**−**			1.04	**2.03**	1.09	1.01	1.62	1.99
1825–1830	1827	Alpha/beta hydrolase fold protein	**<**	**±(+)**	**+(+)**	**+**			1.05	**4.63**	1.36	1.71	1.54	1.61
	1828	Acyl-CoA dehydrogenase type 2	**<**	**+(+)**	**±(±)**	**+**			1.04	**14.83**	**4.63**	**4.56**	**2.87**	1.71
	1829	Rieske-type protein	**<**	**+(+)**	**−(±)**	**±**			1.13	**6.11**	**2.04**	1.95	**2.27**	**2.08**
1847–1852	1848	Putative extradiol dioxygenase	**>**	**−(+)**	**±(±)**	**±**	**3.39**		**3.23**	**28.64**	**2.75**	**3.05**	**3.14**	**2.79**
1860–1861	1861	Dioxygenase motif	**>**	**−(±)**	**±(±)**	**−**			1.54	**6.29**	**2.50**	**3.54**	0.77	1.69
2634–2636	2634	Benzoate 1,2-dioxygenase; alpha	**>**	**+**	**+**	**+**	**28.8**		**5.06**	**2.36**	1.42	1.87	**2.07**	**3.05**
3055–3066	3055	Alpha/beta hydrolase fold protein (*dxnB2*)	**>**	**+(±)**	**−(−)**	**+**			**2.60**	1.65	0.68	0.64	0.85	0.90
	3056	Rieske-type protein; alpha subunit (putative salicylate 5 hydroxylase)	**>**	**+(+)**	**+(+)**	**+**	**2.44**		**2.31**	**2.19**	0.78	0.59	0.73	0.82
	3057	Rieske; beta subunit	**>**	**+(−)**	**−(±)**	**+**			**2.35**	1.07	0.64	0.51	0.57	0.57
	3058	Maleylacetoacetate isomerase″	**>**	**+(+)**	**+(+)**	**+**	**2.42**		**2.48**	0.74	0.55	0.41	0.38	0.45
3083–3084	3084	5-Oxopent-3-ene-1,2,5-tricarboxylate decarboxylase	**<**	**+(+)**	**+(+)**	**+**	**3.88**	**2.74**	1.35	1.46	1.06	1.26	1.39	1.19
3085–3086	3086	Gentisate 1,2-dioxygenase like protein	**>**	**+(+)**	**+(+)**	**+**	**3.92**		1.49	**2.03**	1.32	1.51	1.53	1.07
3087–3096	3087	2,4-Dihydroxyhept-2-ene-1,7-dioic acid aldolase	**<**	**±(+)**	**±(+)**	**±**	**16.9**	**17.71**	0.98	1.01	0.93	**2.06**	1.03	1.53
	3094	Putative extradiol dioxygenase	**>**	**+(+)**	**+(+)**	**+**	**5.33**	**2.79**	1.85	1.72	1.28	0.96	0.93	1.10
3416–3418	3418	Putative extradiol dioxygenase		**+(−)**	**−(±)**	**+**			1.13	**2.95**	1.96	**2.62**	**2.95**	1.82
3863–3865	3865	4-Hydroxyphenylpyruvate dioxygenase	**<**	**+(+)**	**+(+)**	**+**	**7.99**		1.73	1.40	1.60	**2.17**	**2.48**	**3.23**
4273	4273	Vanillate monooxygenase	**>**	**+(+)**	**+(+)**	**+**			1.13	1.20	1.25	1.87	**2.20**	**2.51**
4278	4278	Rieske-type protein; alpha subunit	**<**	**+(+)**	**+(+)**	**+**			1.54	**2.33**	1.89	1.84	**2.04**	1.84
4887–4897	4890	Hydroxyquinol 1,2-dioxygenase (*dxnF*)	**<**	**+(+)**	**+(+)**	**+**			**2.87**	1.17	1.26	0.92	0.88	0.85
	4895	Alpha/beta hydrolase fold	**<**	**+(−)**	**+(±)**	**±**			1.29	0.90	1.16	0.93	1.34	1.40
	4896	Aromatic-ring-hydroxylating dioxygenase (*dxnA2*)	**<**	**+(±)**	**+(±)**	**−**			1.85	0.69	1.46	1.61	1.24	**2.10**
	4897	Ring hydroxylating dioxygenase (*dxnA1*)	**<**	**+(−)**	**+(±)**	**±**		**0.48**	**2.93**	1.52	1.32	1.72	1.39	**2.53**
4902	4902	*dbfB* extradiol dioxygenase	**>**	**+(+)**	**+(+)**	**+**		**0.37**	**4.41**	**2.35**	0.79	0.46	0.46	0.67
4922–4925	4923	4-Hydroxy-2-ketovalerate aldolase	**<**	**+(+)**	**+(+)**	**+**		**0.13**	**11.47**	0.84	0.93	0.57	0.61	0.55
5101–5102	5101	Monooxygenase, FAD binding	**<**	**+(+)**	**+(+)**	**+**	**56.05**		**17.27**	0.80	0.94	0.68	0.52	1.10
	5102	Gentisate 1,2-dioxygenase	**<**	**+(+)**	**+(+)**	**+**	**41.98**		**11.31**	0.82	1.19	1.16	1.19	1.16

*For expanded version, see Table S2 in Supplementary Material*.

*^1^Numbering according to Swit-annotation. Genbank Accession Nrs: NC_009511, NC_009507, and NC_009508*.

*^2^+(+), present in two replicate libraries; **−**(**−**), absent in two replicate libraries; **±**, present at less then one-tenth of the abundance in the starting library*.

*^3^Bold-type setting: statistically significant change (*p* < 0.05) compared to control condition*.

## Results

### Genes implicated in DBF degradation

In order to discover specific genes of RW1 that could be implicated in the response to exposure to DBF, we used a combination of genome-wide expression and transposon insertion screening approaches. An estimated 22,000 independent transposon mutants of RW1 were tested as a library for growth in batch on SAL or DBF medium. After five medium passages, corresponding to 50 generations, the distribution and abundance of transposon insertions in the genome was analyzed by Illumina sequencing and compared to that in the starting library. One hundred and thirty nine genes were specifically absent in the library grown on DBF, but were present by at least 30 transposon reads in the starting library (Table S1 in Supplementary Material). Less stringently, 589 genes were covered by transposon insertions at one-tenth or less in the DBF compared to initial library (Table S1 in Supplementary Material). Among a global annotation of putative catabolic genes in aromatic compound metabolism there were 17 genes missing in the DBF library, 5 of which were not simultaneously absent in the SAL library (Table 1; Table S2 in Supplementary Material), suggesting their unique implication in DBF degradation. One of those was previously recognized (Swit_4896 or *dxnA2*), whereas others have not so far been identified as such (i.e., Swit_1643 for FMN-dependent alpha-hydroxy acid dehydrogenase, Swit_1684 for FAD binding monooxygenase, Swit_1757 for a putative Rieske-motif containing protein, and Swit_1861 for a putative dioxygenase). Transposon insertions in the previously identified genes Swit_4895 (*dnxB*) and Swit_4897 (*dxnA1*) were 10-fold or more underrepresented in the DBF-grown library but not completely absent (Table S2 in Supplementary Material). None of the other genes previously implicated in DBF degradation were absent from the DBF transposon library, suggesting they code for redundant functions. In contrast, transposon insertions were absent in several genes in a cluster for phenylacetate degradation (*paa*, Swit_750–758) in both DBF and SAL-libraries, although these genes are actually expressed to a lower level under such growth conditions (see below). Apart from this cluster, no transposon insertions were detected in a number of other putative catabolic genes after both DBF and SAL growth, e.g., Swit_311 carboxymuconolactone decarboxylase, Swit_893 ferredoxin, Swit_1639–1642 part of enzymes for a *meta*-cleavage pathway, Swit_1759 ferredoxin, Swit_2113 acetaldehyde dehydrogenase, Swit_2251 ferredoxin, or Swit_2292 putative extradiol cleavage enzyme (Table S2 in Supplementary Material). Two of those (Swit_1640, Swit_1641) are 100 and 96% identical, respectively, to Swit_2112 and Swit_2113, and may therefore have been missed because the transposon insertion sites can not be uniquely mapped to one region on the chromosome.

Interestingly, some fifty transposon insertions were at least 10-fold or more abundant in the DBF library compared to time 0, where they were present with at least 30 reads (Table S3 in Supplementary Material), suggesting a small fitness increase of such mutants under those growth conditions. Although this list is still quite long and cannot be interpreted succinctly, it is interesting to find a number of putative catabolic genes, regulatory factors, and stress response factors (Table S3 in Supplementary Material).

### Specific gene expression in presence of DBF

**Figure 1:**
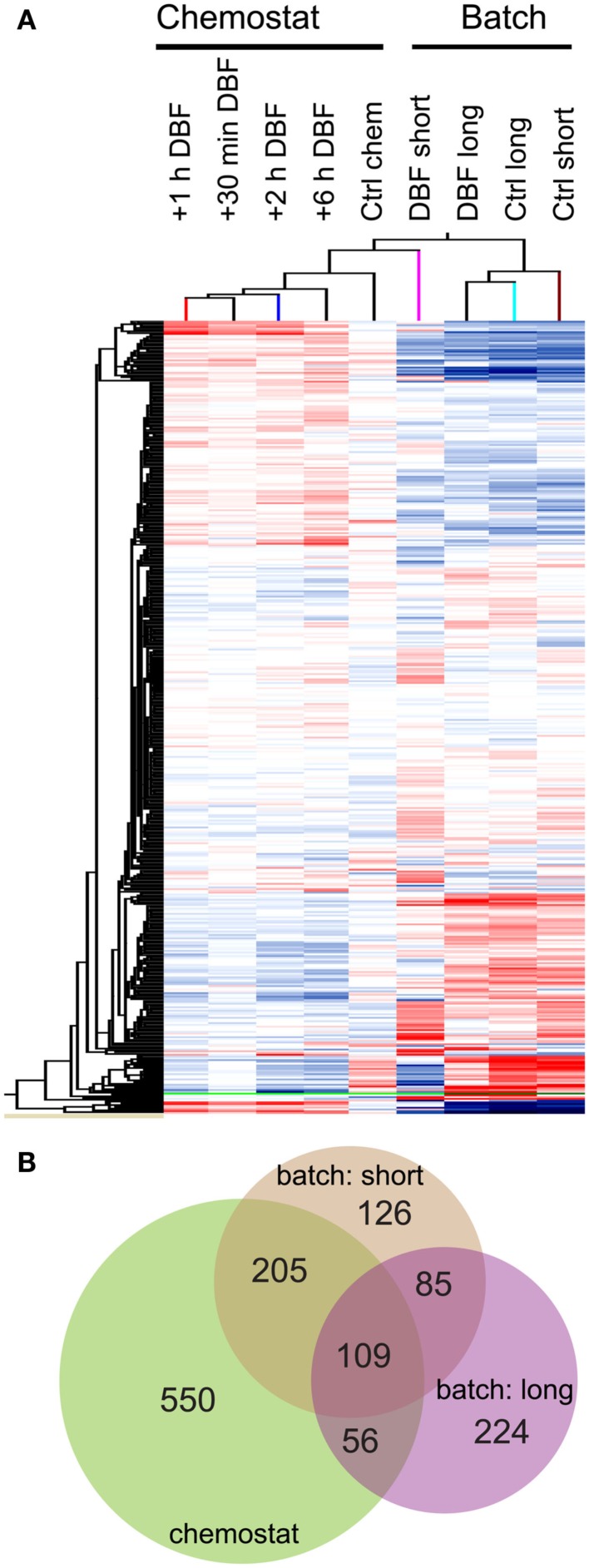
**(A)** Hierarchical clustering of RW1 gene expression over all conditions generated by GENESPRING GX. Short refers to the short DBF exposure in batch cultures, Long to cells grown in batch cultures on DBF, and Chemostat refers to the transient exposure to DBF in continuously grown cultures. Color code: red, increased expression; blue, decreased expression. **(B)** Venn diagram (Hulsen et al., 2008) grouping the genes differentially expressed in the DBF exposure experiments compared to PHE-control conditions. Numbers represent genes exclusive for one condition, while the numbers in the intersections represent those occurring between two or more conditions.

**Figure 2:**
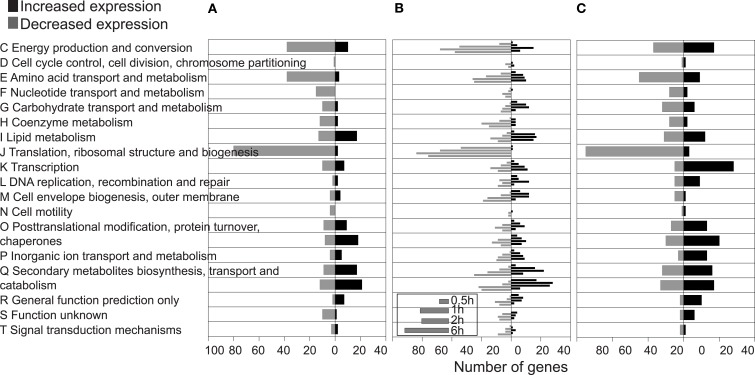
**Proportional abundances of differentially expressed genes in conditions of DBF exposure compared to PHE, categorized per COG**. **(A)** Short exposure to DBF in PHE-grown batch cultures, **(B)**, transient exposure to DBF in chemostats, and **(C)** growth on DBF in batch culture. Note that **(B)** shows data sets from the four time points after addition of DBF to the chemostat (see inset).

In order to further discover genes for DBF metabolism, we used microarray analyses to examine RW1 genome-wide gene expression in the presence or absence of DBF and under a variety of cellular growth conditions. Comparison of all probe signals across all growth conditions showed two broad clusters of expression patterns, roughly representing the differences (almost opposite behavior) of growing in chemostat and in batch culture (Figure 1A). In comparison to PHE-grown cells, 525 RW1 genes were differentially expressed during short exposure to DBF in batch (Figure 1B, File 1 in Supplementary Material), 920 genes in at least one time point of the transient exposure in chemostat (File 1 in Supplementary Material), and 474 genes in the long exposure in batch cultures (File 1 in Supplementary Material). A higher number of genes were shared between the short and transient DBF exposures (205 genes) than among the other conditions. A total of 109 genes were commonly differentially expressed to all the conditions of DBF exposure, among which 18% with increased, and 78% with decreased expression in DBF compared to control (Table S4 in Supplementary Material). Globally speaking, the COG distribution of differentially expressed genes between the three growth conditions with DBF was similar, with notable exceptions of COG-C (*Energy production and conversion*), COG-K (*Transcription*), and COG-N to COG-Q being more abundant in DBF-grown cells (Figure 2).

### Long exposure to DBF in batch cultures

**Figure 3:**
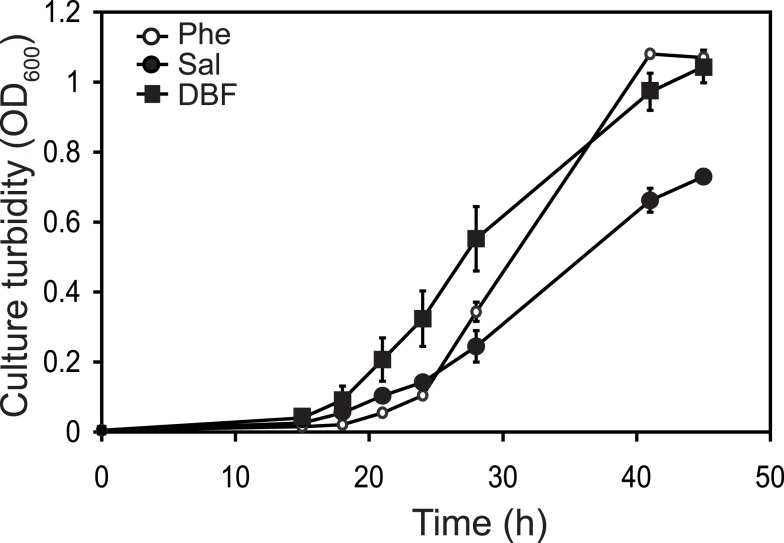
**Growth of *S. wittichii* RW1 on minimal media (MM) with PHE (open circles), SAL (closed circles), or DBF (closed squares) as sole carbon and energy source**.

Maximum specific growth rates of RW1 in batch culture were different on PHE, SAL, or DBF, with 0.24 ± 0.09, 0.16 ± 0.01, and 0.20 ± 0.03 h^−1^, respectively (Figure 3). In comparison, 474 genes were differently expressed between growth on PHE and DBF (8.6% of the whole genome), with 52% having increased and 48% decreased expression (File 1 in Supplementary Material). Exponentially growing cells on SAL showed 231 differentially expressed genes compared to PHE (168 increased and 83 decreased), and 167 between SAL and DBF (87 up and 80 down). Among genes putatively involved in aromatic compound metabolism, most strikingly, the *paa* gene cluster (Swit_748–762) was much lower expressed both during SAL and DBF growth compared to PHE (Table 1; Table S2 in Supplementary Material). Conversely, a set of four closely located gene clusters showed increased expression in SAL (Swit_3083–3094), but not in PHE or DBF. The Swit_3083–3094 clusters may thus constitute the pathway genes specifically expressed during growth on SAL, although none of them appeared to be essential in the transposon scanning (Table S2 in Supplementary Material). By contrast, the plasmid-located *dxn* catabolic genes (between Swit_4897 and Swit_4930) had a decreased expression in SAL compared to DBF, but were overall higher expressed in DBF- than PHE-grown cells. This confirms that they are specifically expressed during growth on DBF. Of these, only Swit_4896 (*dxnA2*) and to a lesser extent Swit_4895 (*dxnB*) and Swit_4897 (*dxnA1*) were essential for growth on DBF (Table S2 in Supplementary Material). Finally, the two plasmid-located genes Swit_5101 and Swit_5102 (coding for a monooxygenase and gentisate 1,2-dioxygenase) had a strongly increased expression during growth on SAL and DBF, compared to growth on PHE. However, again these two genes are not essential for growth on SAL or DBF (Table 1; Table S2 in Supplementary Material).

Other notable differentially expressed genes included those for putative transport functions, such as aquaporin Z (Swit_0028) and a number of TonB-dependent like receptors. For example, the TonB-dependent like receptor DxnC (Swit_4894) was up to six times higher expressed in DBF-grown cells as compared to PHE-grown cells. A putative efflux system encoded by Swit_3219–3222 was expressed up to 50 times higher in SAL- than DBF- or PHE-grown cells (File 1 in Supplementary Material).

### Short exposure to DBF in batch cultures

When RW1 cells were exponentially grown on PHE, after which they were exposed for 30 min to medium with DBF, 525 genes were differentially expressed compared to the control (9% of the whole genome). Sixty three percent of those had decreased and 37% displayed increased expression. A large proportion of differentially expressed genes (171 of 525) consisted of genes grouped in COG-E (*Amino acid metabolism*), COG-C (*Energy production and conversion*), and COG-J (*Translation, ribosomal structure, and biogenesis*, Figure 2A). The expression of most of those (91%) decreased in DBF- compared to PHE-exposed cells, including genes for ribosomal proteins, tricarboxylic acid cycle (TCA), oxidative phosphorylation, tRNA-synthetases, and elongation factors. This suggests temporary growth arrest and a major reconfiguration of the catabolic pathways.

The expession of 17 genes participating in lipid metabolism, including genes from the fatty acid metabolism pathway, were 2- to 16-fold higher in DBF- than PHE-exposed cells. Also, between 13- and 31-fold higher expression was detected of two genes coding for OmpA-domain containing proteins (Swit_1172 and Swit_2322) and of two genes for putative efflux pumps (Swit_3724 and Swit_3725). Four genes involved in cell motility (Swit_1264, Swit_1268, Swit_1270, and Swit_1458) were two to fivefold lower expressed in the presence of DBF (Table 2).

**Table 2:** **RW1 gene cluster representatives with significantly changed expression levels or proportional abundances in transposon libraries**.

Swit Locus	Name	Tn library^1^			Expression, fold change^2^							
		TN01	SAL	DBF	SAL/PHE	SAL/DBF	DBF/PHE	DBF shock	Chemostat shift			
									30 m	1 h	2 h	6 h
**STRESS RESPONSE**												
0016	Redoxin domain containing protein	**+**	**+**	**+**			0.99	1.75	**2.51**	**5.13**	**3.76**	**2.69**
0847	Glutathione S-transferase domain containing protein	**±**	**+**	**+**			**2.50**	1.82	1.89	**2.57**	**2.23**	1.34
2245	Glutathione S-transferase domain containing protein	**+**	**+**	**+**			**3.10**	1.56	**3.16**	**4.72**	**4.11**	**2.28**
3730	Catalase	**+**	**+**	**+**			**206.50**	1.87	1.16	**2.73**	**10.6**	**4.89**
3741	1-Cys peroxiredoxin	**−**	**−**	**−**			**28.84**	1.60	**2.33**	**5.46**	**8.06**	1.67
3743	1-Cys peroxiredoxin	**+**	**+**	**+**			**102.54**	1.83	1.44	**2.89**	**12.7**	**3.12**
3979	ATP-dependent DNA ligase	**+**	**+**	**+**			**2.46**	1.35	**2.39**	**3.61**	**2.45**	1.53
3982	DNA ligase D	**+**	**+**	**+**			**3.25**	1.05	**3.56**	**7.31**	**5.43**	**2.68**
4092	DNA repair protein RadA	**±**	**−**	**±**			1.22	0.89	1.39	1.72	**2.20**	**2.31**
4209	Glutathione-dependent formaldehyde-activating, GFA	**+**	**−**	**+**			**3.56**	1.82	**3.05**	**5.54**	**4.32**	**2.41**
5282	DNA ligase D	**+**	**+**	**+**			**2.20**	1.12	**2.89**	**4.17**	**3.48**	1.93
5311	Catalase	**+**	**±**	**±**			**2.91**	1.27	**4.56**	**6.41**	**4.53**	**2.23**
**DNA METABOLIC PROCESS**												
0001	Chromosomal replication initiator protein DnaA	**−**	**−**	**−**			**0.25**	0.68	**0.40**	**0.30**	**0.23**	**0.31**
2767 = 3050 = 4905 = 5124	IS4 family transposase	**ND**	**ND**	**ND**			**4.24**	**4.44**	1.21	0.90	1.04	1.26
4903	Transposase IS3/IS911 family protein	**ND**	**ND**	**ND**		**0.17**	1.26	**10.50**	0.83	0.51	0.36	0.52
4930	Transposase Tn3 family protein	**+**	**+**	**+**		**0.46**	1.96	1.89	0.95	0.93	1.16	0.96
5075	Transposase Tn3 family protein	**+**	**+**	**+**			**2.50**	**2.00**	1.47	1.33	1.19	1.23
5078	Transposase IS66	**ND**	**ND**	**ND**	**2.14**		**2.48**	**2.33**	0.97	1.05	1.34	1.46
5109	IS4 family transposase	**+**	**+**	**+**			**2.64**	1.99	1.16	1.24	1.16	1.30
**TRANSCRIPTION AND TRANSLATION**												
0431	RNA polymerase sigma factor RpoD	**−**	**−**	**−**			**0.29**	0.54	0.62	0.65	0.63	0.85
1325	Ribosomal protein L17	**+**	**−**	**+**			**0.09**	0.53	**0.35**	**0.25**	**0.19**	**0.13**
1358	Ribosomal protein S12	**−**	**−**	**−**			**0.02**	**0.27**	0.47	**0.34**	**0.23**	**0.20**
3924	ECF subfamily RNA polymerase sigma 24 factor	**+**	**+**	**+**			**10.36**	**2.26**	**4.27**	**4.28**	**2.58**	1.16
3972	ECF subfamily RNA polymerase sigma 24 factor	**−**	**−**	**−**			1.54	1.17	0.92	1.09	1.47	**2.57**
4736	ECF subfamily RNA polymerase sigma 24 factor	**±**	**−**	**+**			1.27	0.94	1.27	1.42	1.49	**2.25**

*^1^+, Present in the library; −, absent in the library; **±**, present at less than one-tenth compared to the starting library; ND, no reads assignable (duplicated genes)*.

*^2^Bold-type setting: statistically significant change (*p* < 0.05) compared to control condition*.

Expression of genes involved in aromatic compound metabolism was clearly different during immediate exposure to DBF as compared to exponential growth on DBF (Table 1; Table S2 in Supplementary Material). Several genes were not as highly expressed as during growth on DBF as sole carbon and energy source, such as Swit_2634–2636 (benzoate dioxygenase), the Swit_3055–3066 cluster, the plasmid-located Swit_4887–4897 cluster (with *dxnF*, *dxnC*, *dxnA1A2*) or Swit_4902 (*dbfB*). More strikingly, expression of Swit_4923, Swit_5101, and Swit_5102 was not detectable at all, whereas these genes were up to 17-fold higher expressed during growth on DBF. In contrast, expression of other genes appeared more clearly after immediate exposure to DBF, such as that of Swit_4278 (uncharacterized aromatic ring dioxygenase), Swit_3418 (putative extradiol dioxygenase), Swit_3086 (putative gentisate 1,2-dioxygenase), Swit_1827–1829, Swit_1848 (putative extradiol dioxygenase), and Swit_1861 (uncharacterized dioxygenase). Also expression of Swit_3793 (membrane transporter for aromatic hydrocarbons) was 21-fold higher in immediate response to DBF. Conversely, and in agreement with growth on DBF alone, expression of the *paa* pathway genes (Swit_748–758) was again much lower than in cells exposed to PHE.

Interestingly, several genes putatively involved in stress response displayed from 18-to 109-fold increased expression immediately after contact to DBF but not during growth on DBF, such as a catalase (Swit_3730) and two 1-cys-peroxiredoxin genes (Swit_3741 and Swit_3743). Also, and this more consistently throughout all growth conditions with DBF, an alternative ECF sigma 24 factor showed up to 10-fold higher expression (Swit_3924, Table 2).

### Transient exposure to DBF in continuous cultures

To avoid the sudden starvation of cells upon transient exposure to DBF as in the batch shock exposure experiment, we grew RW1 in continuous culture with 5 mM PHE and under carbon-limited conditions. A sudden transition to DBF exposure was achieved by spiking the reactor medium instantaneously with saturating DBF amounts (5 mg/l) and simultaneously changing the medium inflow to one containing MM plus 5 mM PHE and saturating DBF. Under these conditions, a total of 920 genes was found to be differentially expressed in at least one time point evaluated (17% of the whole genome) with 154 genes appearing 30 min after exposure to DBF (53% with increased and 47% with decreased expression), 415 at 1 h (51% increased and 49% decreased), 663 at 2 h (38% up and 62% down), and then decreasing to 465 at 6 h (28% up and 72% down). A set of 48 genes expressed similarly across all time points, 2 with increased and 46 with decreased expression (File 1 in Supplementary Material).

A large proportion of differentially expressed genes (204) grouped in COG-C (*Energy production and conversion*), COG-E (*Amino acid transport and metabolism*), and COG-J (*Translation, ribosomal structure, and biogenesis*). 81% of these displayed decreased and 19% increased expression (Figure 2B), suggesting again partial growth arrest but not as severe as in the batch shock exposure (File 1 in Supplementary Material). Interestingly, a clear transition and an adaptive effect could be seen from the abundance of differentially expressed genes in the different COG categories over time after the start of exposure to DBF (Figure 2B). Among the COG-I (*Lipid metabolism*), 37 out of 54 genes increased expression, as well as three genes coding for OmpA-domain containing proteins (Swit_0853, Swit_2278, and Swit_2322). A cluster comprising three genes involved in trehalose synthesis (Swit_3608–3610) temporarily increased expression in response to DBF. Again, several stress response genes, such as for a redoxin, catalases, 1-cys peroxiredoxins, or DNA repair proteins transcribed up to 13 times higher in response to DBF induction in chemostat (Table 2), as well as genes for three putative ECF sigma 24 factors (Swit_3924, Swit_3972, and Swit_4736).

Compared to the transient batch DBF shock and growth on DBF, the shift exposure to DBF in chemostat caused again a somewhat different transcription pattern of genes involved in aromatic compound metabolism (Table 1; Table S2 in Supplementary Material). As examples, increased transcription of the cluster Swit_1827–1830 was detectable but this leveled out from early (30 min) to late transition (6 h). Cluster Swit_1848–1852 and Swit_1861 expressed more like batch growth on DBF but less than after batch shock exposure. Expression of the benzoate dioxygenase (Swit_2634–2636) increased similarly as during batch growth on DBF or after DBF shock. In contrast, expression of the cluster Swit_3055–3066 and of the *dxn* gene cluster (Swit_ 4887–4897) was no longer increased in chemostat (except *dxnA1* after 6 h). In contrast, expression of Swit_3863–3865 was higher in chemostat but not in the two other DBF exposure conditions. Consistent with the other two growth conditions with DBF, expression of the *paa* cluster was lower in chemostat-grown cells exposed to DBF compared to PHE, which was already detectable after 30 min.

## Discussion

The major objective of this work was to study the global response of *S. wittichii* to exposure to DBF. Although DBF functions as a carbon and energy source for *S. wittichii* its behavior is complex, most of all because of the properties of DBF itself and secondly, because of numerous redundancies in aromatic compound catabolic pathways. In order to study this question, we used two different techniques: microarray analysis of genome-wide gene expression (Johnson et al., 2008, 2011) and genome-wide transposon screening (Langridge et al., 2009). At the low aqueous solubility of DBF we expected that the magnitude of direct gene expression difference would be quite small, as normally the compound’s concentration or more precisely, the compound flux, determines the promoter response (Leveau et al., 1999; Tecon et al., 2006). Moreover, gene induction magnitudes of catabolic pathways have been shown to be maximal during transition phases but level out when cells reach a new equilibrium (Leveau et al., 1999). Finally, the inductive response of a carbon source is dependent on the simultaneous presence of other (possibly more preferred) carbon sources (Duetz et al., 1994). We thus designed three different types of exposure of cells to DBF, all of which necessarily compromised one or other aspect of cellular physiology. In the first (named short exposure in batch) RW1 was grown on PHE as sole carbon and energy source into exponential phase in order to have actively growing cells. At the time of exposure, the cells were rapidly harvested and resuspended in the same medium with PHE or with DBF in order to maximize their potential response to the new carbon source. Although numerous genes including catabolic pathway genes increased expression during exposure to DBF compared to PHE, the cells clearly completely changed their physiology and went through a period of growth delay. Gene expression for central metabolic pathways, such as the TCA cycle, amino acid metabolism, but also for ribosomal proteins, elongation factors, tRNA-synthetases, and cell division was immediately declining. This suggests that the cells exposed to DBF during a short period cannot immediately gain sufficient energy from the available DBF in solution and go through a period of starvation and stress response. However, this experimental condition is still important, because it mimics what might happen when cells are inoculated from laboratory-grown culture into a bioremediation site.

In the second type of experiment (transient exposure in chemostat) RW1 cells were growing continuously under carbon limiting conditions with PHE, in order to make sure they would not suffer energy losses during transition. As a consequence of maintaining carbon limiting conditions, the actual PHE concentration in the medium is very low and the cells were expected to react instantaneously to a newly added carbon source (DBF). Indeed, we observed a clear transient response (Figure 2B) implicating a large number of genes, surprising given the controlled conditions of chemostat operation. Even during these controlled growth conditions, the cells experienced DBF not just as a new carbon substrate but rather as a stress factor, necessitating the immediate differential regulation of specific stress response genes. We detected increased expression of genes for catalases, peroxiredoxins, and glutathione-s-transferases, which form a known strategy for the detoxification of xenobiotics and reactive oxygen species (Dominguez-Cuevas et al., 2006; Dayer et al., 2008). Genes implicated in DNA repair, chaperones, and OmpA-domain containing proteins (a putative sensor of membrane integrity) also increased expression. These responses are similar to what has been observed when exposing *P. putida* KT2440, or *P. putida* DOT-T1E to the aromatic hydrocarbons toluene and pentachlorophenol (Segura et al., 2005; Dominguez-Cuevas et al., 2006; Muller et al., 2007). Both the short exposure to DBF in batch cultures and in chemostat also led to specific differential expression of the RpoD sigma factor and of alternative ECF factors (Table 2). ECF sigma 24 factors have been implicated in the response of cells to perturbations such as extracytoplasmic protein miss folding, heat shock, oxidative or solvent stress (Mecsas et al., 1993; de las Penas et al., 1997; Testerman et al., 2002; Dominguez-Cuevas et al., 2006), and could play an important role in the adaptability of *S. wittichii*, the genome of which codes for 14 different putative ECF sigma 24 factors. Previously, Johnson et al. (2011) reported an increase in expression of two ECF sigma 24 genes (Swit_3836 and Swit_3924) in RW1 upon exposure to water potential decrease by NaCl, the expression of one of which (Swit_3924) also increased during shock exposures of cells to DBF (Table 2). In a similar way, *B. xenovorans* LB400 and *P. putida* KT2440 have been found to induce ECF sigma 24 in response to hydrocarbons such as benzoate and toluene (Denef et al., 2006; Dominguez-Cuevas et al., 2006).

In the third experiment (long exposure in batch) RW1 cells are grown in batch either on PHE, SAL, or on DBF as sole carbon source, and in all cases harvested at the same culture turbidity in exponential phase. Stress response gene transcription under those conditions was not different between DBF or PHE-grown cells, indicating that the cells adapt after a while to metabolize DBF without stress.

**Figure 4:**
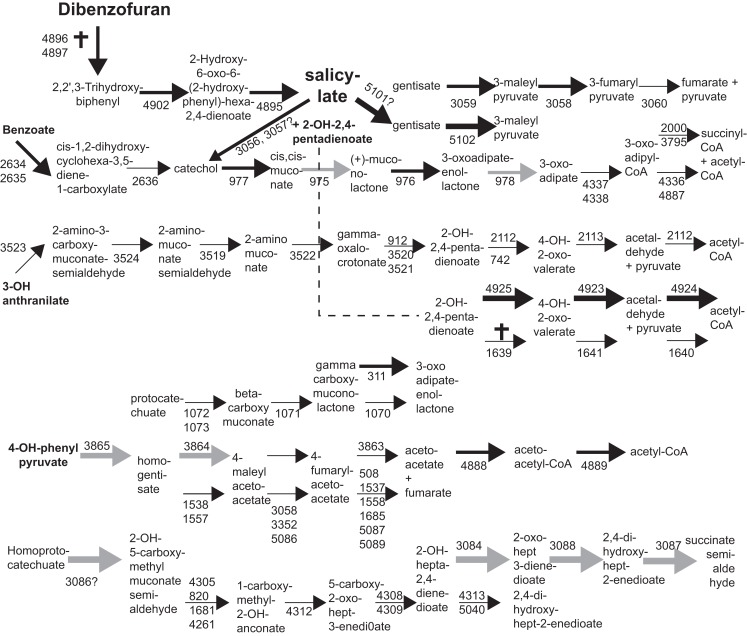
**Compilation of possible encoded aromatic compound degradation pathways in *S. wittichii* RW1 with relevance to either DBF or SAL metabolism**. Numbers below arrows correspond to gene names (e.g., Swit_5101). Thick arrows indicate gene induction during growth on SAL or DBF compared to PHE. Gray arrows point to induction on SAL only. Crosses indicate genes in which no transposon insertions were found after 50 generations growth on DBF. Pathway predictions were done using KEGG and NCBI.

Perhaps surprisingly, in the light of typical linear pathway thinking from work on catabolic pathways in pseudomonads, expression of pathways for aromatic compound metabolism even with a single compound (DBF) involved a myriad of possibilities. This suggests, first of all, that *S. wittichii* has an extreme redundancy in its use of aromatic compound metabolism (Figure 4). This was evident from the finding that actually very few transposon insertions completely abolished growth on DBF (Table 1; Table S2 in Supplementary Material). Secondly, the pathways seem to operate under distinct growth conditions and, not unlikely, in temporarily different stages (Table 1; Table S2 in Supplementary Material). Among the few exceptions to this were two clusters (Swit_1847–1852, Swit_1860–1861), to a lesser extent Swit_2634–2636 (benzoate dioxygenase), and Swit_3864–3866 (homogentisate dioxygenase) that were always expressed higher in presence of DBF, irrespective of the growth conditions. In contrast, increased expression of the typical *dxn* genes discovered so far was only detectable during growth on DBF, but not in the two other conditions. This situation of pathway parallelism is analogous to the existence of three different pathways for benzoate metabolism in *Burkholderia xenovorans*, that can operate under different conditions in degradation of (polychlorinated) biphenyls and chlorobenzoates (Denef et al., 2006).

Which genes can we finally conclude are “implicated” in DBF degradation? Although SAL has been postulated as an intermediate for DBF degradation, the pathway that is most highly and specifically expressed during SAL growth (Swit_3086–3095) is not particularly induced during DBF growth (Table 1; Figure 4; Table S2 in Supplementary Material). More likely are two pathway branches that would lead to gentisate from SAL (Swit_5101/5102) or to catechol (Swit_3056/3057, Figure 4). Both these gene groups are induced on DBF, although their precise function cannot be sufficiently predicted from sequence comparisons only. The other major metabolite formed from DBF is 2-hydroxy-2,4-pentadienoate, which could be further processed to acetyl-CoA through activity of the enzymes encoded by the Swit_4923–4925 cluster (Figure 4). Although Swit_4923–4925 were higher transcribed on DBF but not on SAL, they do not seem to be essential for growth on DBF, since transposon insertions were detected in all of them. At least three alternative pathways seem to be encoded on the RW1 genome (Figure 4), and, interestingly, transposon insertions in one of those genes (Swit_1639) were never recovered. This suggests that the Swit_1639–1641 pathway is somehow important, although the genes are not specifically transcribed to higher levels during growth on DBF compared to SAL or PHE. It is also possible that bioinformatic functionality predictions here are incorrect because of confusion and close sequence homology between the paralogous enzymes 4-oxalocrotonate-decarboxylase and 2-hydroxy-2,4-pentadienoate hydratase (Figure 4). Growth on SAL alone leads to increased expression of a different set of metabolic pathway genes than on DBF, suggesting different intermediate processing via homoprotocatechuate and/or 4-hydroxyphenylpyruvate (Figure 4). Such pathways, however, are currently incomplete with no known links between SAL and homoprotocatechuate.

In conclusion, we find that the DBF “metabolome” in *S. wittichii* is surprisingly complex with numerous parallel and redundant branches. The strategy of parallelism and redundancy may have specific ecological advantages in certain niches with perhaps multiple structurally similar substrates, but may be too costly to maintain competitively in environments with abundance of singular substrates that may favor bacteria with single pathway induction. Possibly, the parallelism and redundancy suggested by our “population” data suggest are in reality split across individual cells with a high degree of metabolic heterogeneity.

## Conflict of Interest Statement

The authors declare that the research was conducted in the absence of any commercial or financial relationships that could be construed as a potential conflict of interest.

## Supplementary Material

The Supplementary Material for this article can be found online at http://www.frontiersin.org/Microbiotechnology,_Ecotoxicology_and_Bioremediation/10.3389/fmicb.2012.00300/abstract

## Footnotes

^1^https://depts.washington.edu/cshlab/html/rnaseq.html

^2^http://www.ncbi.nlm.nih.gov/pubmed/11726920
